# Co-designing a digital health resource for transgender and gender diverse older adults

**DOI:** 10.3389/fpubh.2026.1919463

**Published:** 2026-10-28

**Authors:** Lauren Catlett, Crystal Chu, Noelle Marie Javier, Kimberly D. Acquaviva

**Affiliations:** 1 School of Nursing, University of Virginia, Charlottesville, VA, United States; 2 Mount Sinai Icahn School of Medicine, New York, NY, United States

**Keywords:** co-design, digital health, gender diverse, gender identity, healthcare, human-centered design, older adults, transgender

## Abstract

**Introduction:**

Transgender and gender diverse (TGD) older adults in the United States (U.S.) face barriers to accessing health information and resources they need to make informed healthcare decisions. More resources are needed to promote their agency and preparedness to navigate an increasingly complex healthcare system and sociopolitical landscape. The purpose of this study was to develop a testable web-based resource to support informed healthcare decision-making for TGD older adults.

**Methods:**

This multimethod co-design study integrated a human-centered design approach that centered the perspectives of TGD older adults in the research process. In the prototyping and testing phases of the study, TGD adults and multidisciplinary professionals from across the U.S. were enrolled to co-develop a web-based resource with the research team during individual and paired interviews. Participants also completed a demographic questionnaire and a website usability survey. Thematic analysis of qualitative data was used to identify design themes, which guided iterative prototyping of the web-based resource. Quantitative data were analyzed descriptively. Complementary data from the usability survey and participant interviews were integrated in a joint display.

**Results:**

A total of 27 participants, of whom 14 (52%) were TGD older adults, enrolled in the prototyping and testing phases. Analysis of qualitative data across both phases yielded five design themes that structured the development of a functional web-based resource: (1) Facilitating healthcare decisions through information and resources; (2) Organizing complex content with clarity and inclusivity; (3) Maintaining security, accessibility, and navigability; (4) Phasing rollout to communities and organizations; and (5) Planning for leadership, stewardship, and partnership. In the prototyping phase, three low- and mid-fidelity prototypes of a health information and resources website for TGD older adults were developed. In the testing phase, participants trialed a mid-fidelity prototype of the website, and their recommendations for improvements were integrated into a high-fidelity prototype for dissemination.

**Conclusion:**

This study is one of few studies using participatory methods to engage TGD older adults in public health research. Through the development of a health information and resources website, this study demonstrates an innovative and feasible community-based co-design approach to addressing barriers affecting healthcare decision-making for TGD older adults.

## Introduction

Increasing complexity in the United States (U.S.) healthcare system compounded by multilevel stigma and health inequities creates considerable barriers to equitable and informed healthcare decision-making for transgender and gender diverse (TGD) older adults (1, 2). Accounting for over 10% of TGD Americans, TGD older adults may be defined as persons aged 50 years old or older whose gender identity differs from the one assigned to them at birth and/or whose gender identity does not conform to societal gender norms (3–5). Barriers to making informed healthcare decisions exist in the TGD community due to factors such as limited research on transgender health for adults, older adults, and minoritized racial groups; inconsistent collection of health data on sexual orientation and gender identity; and policies that limit access to health information and resources (6–8). For example, following a series of executive orders issued by the U.S. presidential administration in 2025, TGD-focused content was removed from key sources of public health information, including the National Institutes of Health and the Centers for Disease Control and Prevention (9). Such political and legislative actions reduce access to health information that may subsequently reinforce poor health outcomes within TGD communities in the long-term (10).

Despite the barriers, TGD older adults have demonstrated key strengths that may be protective in healthcare settings and may promote agency in decision-making. Personal characteristics like perseverance and resourcefulness help TGD older adults navigate increasingly complex health systems (2, 11, 12). TGD community networks have provided information about treatments and procedures that have been largely absent from standard medical sources, and these networks have been a source of care and support for community members with health conditions (2, 13). Personal strengths, social networks, and the drive to thrive are individual- and community-level protective factors that may support healthcare decision-making among TGD older adults.

One form of healthcare decision-making that offers further protection for TGD older adults is advance care planning (ACP), “a process that supports adults at any age or state of health in understanding and sharing their personal values, life goals, and preferences regarding future medical care.” (14) ACP engagement is low in the general population, and it may be lower among TGD older adults, due in part to fears that their wishes will not be honored by healthcare providers (15, 16). However, ACP carries benefits for TGD older adults, such as the ability to name a surrogate decision-maker, especially when a person’s default surrogate decision-maker, or next of kin, does not accept their gender identity (17). As in the general population, greater access to ACP information and resources for TGD older adults is needed to support healthcare decision-making during serious illness and at the end of life (EOL).

An emerging body of evidence has revealed gaps in knowledge about healthcare and EOL care for TGD older adults (11, 18). Recommended approaches to address the gaps include participatory research to initiate systemic change and meaningful involvement of the LGBTQIA+ (lesbian, gay, bisexual, transgender, queer/questioning, intersex, asexual/agender plus other sexual orientations and gender identities) community throughout the research process (18). Research is needed to support the creation of health resources for TGD older adults that would address challenges they face by engaging their strengths, experiences, and knowledge in a participatory design process. In response to the need for research and resources on health and healthcare for TGD older adults, the purpose of this multimethod study was to develop a testable web-based resource to support informed healthcare decision-making for TGD older adults using a human-centered design approach.

## Materials and methods

The analysis that follows draws from data collected during two phases of the study Shaping Affirming Futures with Elders of all gender experiences (SAFEage). In this study, researchers and TGD older adults co-designed an advance care planning resource document and health information website to promote informed healthcare decision-making. This study was approved by the University of Virginia Institutional Review Board for the Social and Behavioral Sciences (IRB-SBS#6104). All participants gave informed consent prior to data collection and were offered $50 per interview as compensation for their participation.

In this multimethod co-design study, a human-centered design (HCD) approach was used to engage TGD older adults in the process of creating a healthcare resource to benefit their communities (19, 20). HCD has five processes: empathizing, defining the problem, ideating, prototyping, and testing (19). The initial three processes were completed in an earlier part of the SAFEage study (21). Consequently, this analysis centers on the prototyping and testing processes (Figure 1).

**Figure 1:**
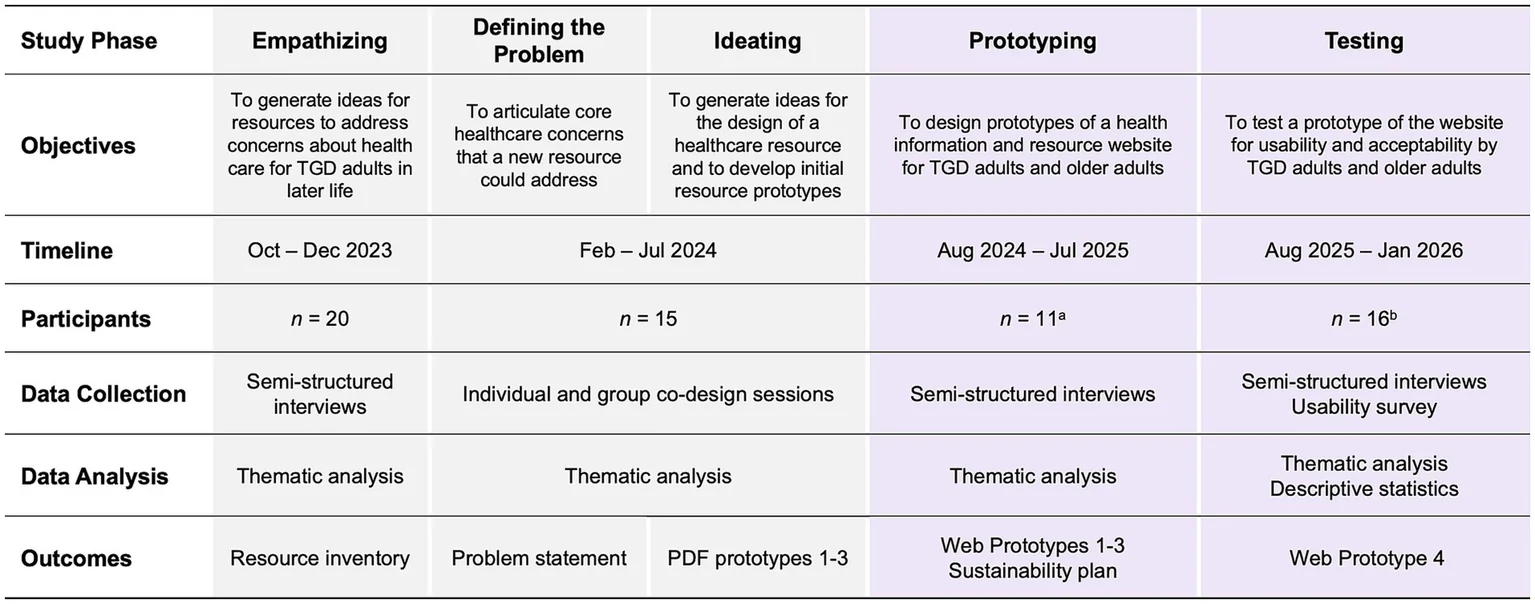
Study design: Shaping Affirming Futures with Elders of all gender experiences. Data from study phases marked in purple were used in this analysis. ^a^Prototyping participants included five (5) TGD older adults in the community and six (6) professionals of diverse gender identities specializing in healthcare, aging services, and design. ^b^Testing participants included nine (9) TGD older adults (aged 50 and older) and seven (7) TGD younger adults (aged 18–49).

Prototyping involves the iterative development of models, or prototypes, to refine the design of a new product or process, while testing is used to assess prototype usability, acceptability, and effectiveness. Prototypes may be classified as low-, mid-, and high-fidelity to indicate how closely the prototype matches the desired product design (22). Prototypes of all three classifications were generated in this study.

### Recruitment and data collection

#### Prototyping phase

In the prototyping phase of the SAFEage study, participants were purposefully selected by the research team for their lived experiences and expertise relevant to the design of a healthcare website for TGD older adults. Prospective participants were eligible for the prototyping phase if they (a) had lived experience as a TGD person or expertise related to healthcare, LGBTQIA+ health, aging, and/or website design; (b) were 18 years old or older (adults of any age were included to allow for professional perspectives in the design process); (c) were living in the U.S.; and (d) had the capacity to give informed consent. Researchers identified eligible persons from (a) existing professional connections; (b) recommendations from community advisors; and (c) participants enrolled in earlier phases of the study. Prospective participants were contacted by email with a formal invitation letter. Those interested in joining the study received a follow-up message with links to the electronic informed consent form and demographic survey to enroll. Participant group size in this phase was determined based on findings from prior research which suggests that expert prototype review is best undertaken by small groups of 5–6 people (23). However, for this study, a larger group size (*n* = 11) was needed to ensure representation of diverse areas of expertise for prototype development.

During initial interviews conducted between August and September 2024, participants reviewed a low fidelity prototype of the website (P1) created by the research team based on findings from the earlier ideating phase of the study. These semi-structured interviews lasted 62 min on average and were conducted by video conference (*n* = 10) and in person (*n* = 1) in private residences or workplaces. Consistent with prior research on the use of co-design with older adults in digital settings, we relied on video conference to include and engage participants who were living at a significant distance from the research team (24). In February 2025, researchers followed up with participants during virtual and in-person individual interviews (*n* = 5 lasting 66.4 min on average) and paired interviews (*n* = 3 lasting 68.7 min on average) to review a mid-fidelity prototype of the website (P2) created by the research team using a cloud-based web builder. Paired interviews were offered as an option to stimulate brainstorming through dialog between participants. All interviews were audio- and video-recorded. See interview guides for both initial and follow-up sessions in Supplementary material S1.

#### Testing phase

Participants in the testing phase were recruited purposefully through national outreach to previously established community connections and LGBTQIA+ organizations serving racially and ethnically diverse populations of TGD adults and older adults. The research team conducted outreach using email messages and electronic informational flyers. Prospective participants completed an electronic screening and interest form using a link on the outreach flyer to determine eligibility. Individuals were eligible for the testing phase if they (a) identified as a TGD adult; (b) were 18 years old or older; (c) were living in the U.S.; and (d) had the capacity to give informed consent. The research team centered outreach on TGD adults aged 50 and older; however, adults of any age were included to invite intergenerational perspectives in the design process. Eligible participants received a follow-up message with links to the electronic informed consent form and demographic survey to enroll. Preliminary participant group size estimates were based on prior usability testing research (25, 26).

Virtual interviews lasting 55.1 min on average were conducted in August and September 2025 to record participant perspectives about the usability and acceptability of a second mid-fidelity prototype of the website (P3). Participants joined their interviews from private and semi-private locations. During the interview, participants were provided a password-protected link to view P3 on a computer, tablet, or mobile phone. Using the “think aloud” approach, interviewers prompted participants to verbalize their perspectives in real time as they explored P3 (27). For two participants who were unable to access P3 on their device, the interviewer shared their screen and navigated through the site at the direction of the participant. Interviews were either audio- or video-recorded based on participant preferences. For the testing phase interview guide, see Supplementary material S2.

Participants in both the prototyping and testing phases completed the Standardized User Experience Percentile Rank Questionnaire (SUPR-Q) (28). The SUPR-Q is an 8-item tool to assess websites on four factors: usability (ease of use and navigation); credibility (perceived trustworthiness and safety); appearance (attractiveness and simplicity of design); and loyalty (likelihood of returning to the website and recommending it to others). After viewing P3, participants completed the SUPR-Q electronically either independently or with the interviewer. Both qualitative and quantitative data informed the development of a fourth prototype (P4).

#### Participants

Because participants contributed substantive design ideas that were essential to co-designing prototypes in each phase, we recognize them as key collaborators in this study. Participant demographic details appear in the Results section and in Table 1. Initials used to refer to participants in the Results are pseudonyms.

**Table 1:** Participant demographic characteristics.

Characteristic	Prototyping	Testing	Both phases
	*n* (%)	*n* (%)	*n* (%)
Participants	11 (100)	16 (100)	27 (100)
Age			
50–76	6 (55)	9 (56)	15 (55.5)
25–49	5 (45)	7 (44)	12 (44.5)
Region			
Midwest (IL, IN, MO)	1 (9)	2 (12.5)	3 (11)
Northeast (NY, NJ, PA)	5 (46)	2 (12.5)	7 (26)
West (AZ, CA)	1 (9)	8 (50)	9 (33)
South (VA, SC, FL)	4 (36)	4 (25)	8 (30)
Gender identity			
Agender	0 (0)	1 (6)	1 (3.5)
Female^+^	5 (46)	2 (12.5)	7 (26)
Gender nonconforming/Nonbinary	3 (27)	7 (44)	10 (37)
Male	1 (9)	2 (12.5)	3 (11)
Trans female/trans woman	5 (46)	5 (31)	10 (37)
Trans male/trans man	1 (9)	5 (31)	6 (22)
Two spirit	1 (9)	0 (0)	1 (3.5)
Another gender identity^a^	1 (9)	1 (6)	2 (7)
Sexual orientation			
Asexual	1 (9)	3 (19)	4 (15)
Bisexual	0 (0)	1 (6)	1 (3.5)
Gay	0 (0)	2 (12.5)	2 (7)
Lesbian	1 (9)	2 (12.5)	3 (11)
Queer	2 (18)	5 (31)	7 (26)
Straight/Heterosexual	7 (64)	2 (12.5)	9 (33)
Another sexual orientation^b^	2 (18)	4 (26)	6 (22)
Race and Ethnicity			
Asian/Asian American	1 (9)	4 (25)	5 (18.5)
Biracial/Multiracial	1 (9)	2 (12.5)	3 (11)
Black/African American	4 (36)	5 (31)	9 (33)
Native Hawaiian/Pacific Islander	0 (0)	1 (6)	1 (3.5)
White/European American	6 (55)	5 (31)	11 (40.5)
Declined to answer	0 (0)	1 (6)	1 (3.5)
Highest degree			
High School Diploma	0 (0)	2 (12.5)	2 (7)
Associates	1 (9)	4 (25)	5 (18.5)
Bachelors	1 (9)	4 (25)	5 (18.5)
Masters	5 (46)	3 (19)	8 (30)
Doctorate	3 (27)	2 (12.5)	5 (18.5)
Other^c^	1 (9)	1 (6)	2 (7)

Total percentages for gender identity, sexual orientation, and race and ethnicity may exceed 100% because participants could select more than one option. ^a^“Trans-femme,” “genderfluid,” “genderqueer”. ^b^“Demisexual,” “pansexual,” “same-gender-loving”. ^c^“Terminal professional master’s degree,” “some college”. ^+^Includes participants who identify as cisgender (*n* = 2).

#### Community advisors

Three transgender older adults served as community advisors for this study. This group of advisors comprised one Black transgender man, one White transgender man, and one White transgender woman, all of whom had lived experiences and community connections relevant to the study. In their role as advisors, they assisted with community outreach during recruitment, offered feedback on prototype drafts, and provided guidance on decision-making.

#### Research team

Researchers involved in data analysis for the SAFEage study included L. C., a White, nonbinary person, postdoctoral researcher in nursing, and principal investigator; K. A., a White, cisgender, queer-identified woman and professor of nursing; C. C., a White, cisgender woman and assistant professor of nursing; N. M. J., an Asian Filipina transgender woman and professor of medicine; and V. L., a White, cisgender woman and associate professor of nursing. The research team had scholarly and clinical expertise in gerontological nursing, palliative care, transgender health, and healthcare decision-making. L. C. had additional experience with web and graphic design.

L. C. had existing professional connections with six participants in the prototyping phase and with one participant in the testing phase. As the interviewer in both phases, L. C. established rapport with participants through shared lived experiences as members of the TGD community and by creating a safe space for open expression through active listening and affirmation. L. C. mitigated any imbalances of power and privilege by approaching each interview with humility and engaging with participants as collaborators during their interviews and during analysis.

### Data analysis

#### Qualitative data analysis

Qualitative data from the prototyping and testing phases were analyzed thematically by the research team to generate a set of design themes describing the prototyping process (29). Deductive codes were applied in the qualitative analysis platform Dedoose (v.10.0.59) from an existing framework used to inform the design of a healthcare resource for TGD adults (21). L. C. used inductive coding to identify additional elements of the framework relevant to web design. K. A., C. C., and V. L. checked code applications for 21% of transcripts from both phases, and L. C. adjusted the codebook and coding applications in the remaining transcripts accordingly. From these codes, an adapted framework of design themes was formed and verified by the research team. These themes describe key design elements that structured prototype development.

Each prototype served as an analytic interpretation of participant ideas shared during their interviews. L. C. used inductive coding in Dedoose to identify design ideas in each transcript and organized those codes by categories to form a comprehensive design checklist for each prototype. L. C. annotated the checklist with practical methods for incorporating each design idea. These methods were reviewed by two other team members (K. A. and V. L.) before being applied to each new prototype. L. C. created P1 in PowerPoint and built P2, P3, and P4 using the cloud-based web building platform Wix. Prototype content was reviewed by TGD older adults and experts in healthcare, social work, and public policy. Member-checking of P1, P2, and P3 occurred during participant interviews, and participants from both the prototyping and testing phases had the opportunity to review and offer feedback on P4. A list of participant ideas related to resource expansion and sustainability was also developed.

#### Quantitative data analysis

SUPR-Q data about the usability of P3 were analyzed descriptively in Excel by calculating mean scores for each factor and for the full set of factors. Each factor has two items, and all items were scored on a 5-point Likert scale except Item 8 which was scored out of 10. Per protocol for this survey, the score for Item 8 was halved before the mean scores were computed. Although a percentile rank score can be computed to compare websites, only the raw SUPR-Q (mean) score was used in this study. Mean scores provided a quantitative estimation of P3 prototype usability and acceptability. Quantitative findings were integrated with qualitative data from the testing interviews in a joint display to illustrate key areas for improvement to address in the design of P4.

### Trustworthiness

Researchers promoted trustworthiness of study findings by recording field notes for each interview, writing analytic and reflexive memos, maintaining a log of analytic decisions, and inviting iterative review of findings by team members, community advisors, participants, designers, and content experts. Because language and narrative were not the primary focus of this study, full transcripts were not returned to participants for review. Instead, to support credibility of the analysis, participants were invited to review aggregate findings (prototypes) that had been generated from the transcripts of their interviews. Including representative participant groups in each phase enhanced the transferability of findings for people of diverse gender identities and racial and ethnic backgrounds from all regions of the U.S.

## Results

A total of 27 participants were enrolled in this part of the study, with 11 in the prototyping phase and 16 in the testing phase. All 11 participants who were invited to the prototyping phase enrolled in the study. Originally, 17 participants were enrolled in the testing phase, but one participant withdrew prior to their interview for personal reasons. Fourteen participants (52%) were TGD older adults, and the remaining participants were either TGD adults under 50 years old (*n* = 11; 40.5%) or cisgender adults of any age who were experts in healthcare or design (*n* = 2; 7.5%). Participant ages across both phases had a mean of 52.2 years and ranged from 25 to 76 years; among older adults, the mean age was 63.4 years. Nine prototyping phase participants and all testing phase participants (*n* = 16) identified as transgender or gender diverse. Across both phases, 11 participants (40.5%) were White or European American, nine (33%) were Black or African American, five (18.5%) were Asian American, three (11%) were biracial, one was Native Hawaiian, and one declined to answer (participants could select more than one race or ethnicity). About two-thirds of participants were living in the southern (*n* = 8; 30%) and western regions (*n* = 9; 33%) of the U.S. Detailed demographic characteristics are reported in Table 1.

The level of education in the prototyping phase was high (73% of participants held master’s or doctoral degrees). Prototyping phase participants had occupational expertise in graphic design (*n* = 1), website design and entrepreneurship (*n* = 1), higher education administration (*n* = 2), aging services administration (*n* = 1), hospice and palliative medicine (*n* = 2), health research (*n* = 2), spiritual ministry (*n* = 1), and financial services (*n* = 1).

When asked about their technology use, 13 of 16 testing phase participants (81.5%) reported that they used websites often or very often, and 11 participants (69%) were very comfortable using websites. Regarding the devices they used to access a website, 15 participants (94%) selected smartphone, 12 participants (75%) selected computer or laptop, and 5 participants (31%) selected tablet (participants could select more than one device).

### Design themes

Five design themes were generated using aggregated data from the prototyping and testing phases. Design themes were numbered 1 through 5 and were listed in a chronological sequence from early to late stages of resource development (Table 2).

**Table 2:** Design principles and themes guiding development of a web-based health resource for TGD older adults.

#	Design principle	Design theme
1	Motivation	Facilitating healthcare decisions through information and resources
2	Composition	Organizing complex content with clarity and inclusivity
3	Interaction	Maintaining security, accessibility, and navigability
4	Dissemination	Phasing rollout to communities and organizations
5	Expansion	Planning for leadership, stewardship, and partnership

Design themes 1, 2, 4 and 5 align with four principles—*motivation, composition, dissemination*, and *expansion—*from a design framework developed earlier in the study (21). Because some data from the prototyping and testing phases did not fit within the earlier framework, a new principle, *interaction*, was added that corresponds to theme 3. These design principles and themes were used to structure prototype development. The following descriptions summarize the main components of each theme.

#### Motivation: facilitating healthcare decisions through information and resources

Participants envisioned the website as a centralized location that would make health information and resources more accessible to TGD older adults. Participant IA described what other participants referred to as a resource “hub”:

And it’s also good that all these different topics are in one place, one location. So, that’s beneficial as well. You do not have to go look all around for them also. And it’s also really good that they tell you what each topic is, and how they can help you as well.

Participants further described the website as a form of support for informed healthcare decision-making. Describing the resource as a catalyst for advance care planning, Participant CZ stated, “I have been negligent in my planning. I should have an advance directive, and I don’t. I started on it, but I didn’t finish it, so [this] website is motivating me to get with it”.

#### Composition: organizing complex content with clarity and inclusivity

Participants stressed the importance of a clear and inclusive site structure. They gave recommendations for the order of pages in the navigation menu and the categorization of external links on the resources page. Reviewing the site in the testing phase, Participant SA commented, “And I think I like how it was organized. Every page is kind of like this: general rights, advocacy, care planning, long-term, end of life, all of these specific questions that I might have are addressed in a way that I could access them”. Participants emphasized the need for a simple design and clearly stated purpose for the website. In the layout and graphics of the site, participants reiterated the need for visible representation of people from diverse racial and ethnic backgrounds:

Somehow, any minorities that do use your site should be made to feel welcome there, too. In my mind, gender orientation and gender identity are inclusive of all ethnicities, but many within non-White groups do not always feel like they are included, as I have been told by some of them…I bet there are ways to make them feel like they are included in other areas of the site, too. (Participant EI).

#### Interaction: maintaining security, accessibility, and navigability

Interaction refers to the user experience of interfacing with the website. Security of user data during site interactions was a requisite feature, as participant MP explained:

Because of privacy concerns and persecution concerns, making sure that, from a data security point of view, it’s really strong. Making sure that no information on there could hurt the trans community or trans individuals.

Participants envisioned a website that was not only secure but also accessible, opposing the idea of password protected entry into the site which could limit access to site resources for some TGD older adults. Site adaptations to promote accessibility for non-English speakers, people with disabilities, people with socioeconomic constraints, and people with different levels of education and learning styles were recommended. To aid site users as they navigate the site, participants proposed features such as a site map and search bar, quick loading time, fewer ‘clicks’ to access site content, and simple navigation aids like ‘breadcrumbs’ that show a user’s location within a website’s hierarchy. Participant CA said, “When we’re looking for information, we just want something, like, go right to it, and you can find the answers”.

#### Dissemination: phasing rollout to communities and organizations

Connecting TGD older adults, healthcare providers, and care partners to the website was a priority for participants. Their recommendations for outreach included providing community education, giving presentations, hosting events, broadcasting the resource through public media, distributing printed materials, and contacting organizations and social networks. Although participants advocated for broad outreach to increase access, they expressed concerns about disseminating at a rapid pace with high visibility in an anti-transgender political climate. Participant AK cautioned, “You don’t want to go viral, but you do want to be able to help people… If you can plant the seeds, then you can let it grow. But it’s figuring out the quiet ways you can plant the seeds”. Recognizing this concern alongside the urgency to make the resource available as soon as possible, a phased dissemination plan was developed: “Just think of phasing it that way, because I think people do need the resource… just thinking through what could feasibly come out sooner than later” (Participant EF).

#### Expansion: planning for leadership, stewardship, and partnership

Reflecting on future growth of the resource, participants commented on the need for leadership to sustain the expansion. Participant CD discussed the benefits of forming an advisory committee and nonprofit organization:

And then really kind of either form a committee, strange as that sounds, or, you know, depending on who you are getting involved for the longer term, get everybody signed up to do one of those activities, and just kind of run it like a mini organization, almost.

Participant MW called for expansion of the leadership team for sustaining the resource over time: “It shouldn’t be reliant on the efforts of a person or a small group of individual people because that tends to be how we see nonprofits die”.

Site maintenance was the priority for ongoing stewardship of the resource, as articulated by Participant CZ: “Sometimes when I’ve gone to a page of resources like this, people don’t keep them updated. And so, I end up wasting a lot of time trying to get in touch with an organization that’s not there”. To increase access and buy-in, participants called for establishing partnerships with aging organizations, medical professional organizations, healthcare agencies, conference organizers, and others. Recommendations for resource expansion were documented in a sustainability plan endorsed by prototyping phase participants.

### Prototyping phase

#### Prototype 1

With data from 19 co-design and member-checking sessions (individual and paired interviews) during the prototyping phase, three prototypes of the website were developed. The first prototype (P1) was a low fidelity mock-up of a website homepage (see Figure 2A) whose content and layout were modeled on suggestions from participants in earlier phases of the study. Participant ideas, insights, and recommendations are categorized by design principle (see Table 2) in the following sections.

**Figure 2:**
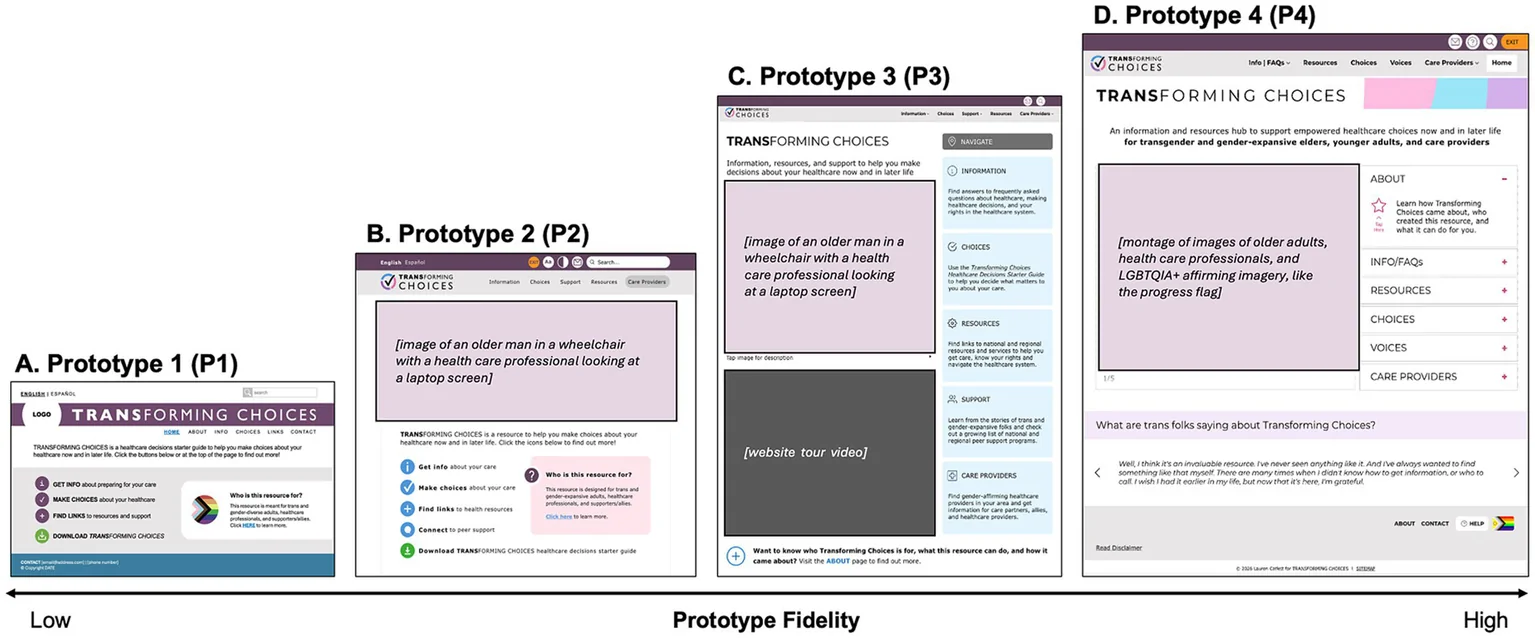
Development of prototypes for a web-based health resource for TGD older adults.

##### Design Principles 2 and 3 (composition and interaction)

Prototyping phase participants assessed P1 and suggested modifications during their interviews. Participant CD emphasized that the homepage should appeal to an older audience:

I think [for] the website you would want some sort of front end on it that’s much more website-like, quite frankly, [that] looks less like a resource guide, and looks more like just a splash page…that says something to the effect of: ‘Older adults, you have questions and you’re questioning your gender identity?’ Something that just really invites them to start taking a look.

Accessibility for an older audience was a priority for participants in the prototyping phase. Participant TI explained the need for adjustments to P1 to accommodate older website users with low vision:

Now you see how I’m doing this right? [Participant TI squints and moves closer and farther from the screen] I’m a young elder, and I’m learning how to wear glasses…And being online sometimes is frustrating for me now because I cannot always see it.

##### Design principle 1 (motivation)

Additional recommendations for the next iteration of the prototype included maintaining focus on the intended audience throughout the website; clarifying gender-related and health-related terms for an older audience who might be unfamiliar with them; and providing a “visual that’s absolutely identifiable with being queer and being old” (Participant LH).

#### Prototype 2

The second prototype (P2), a mid-fidelity model of the website, was developed for participants to visualize the site’s layout (see Figure 2B).

##### Design principle 2 (composition)

The P2 homepage included a slideshow of images of older adults and an image of the progress flag. Participants called for greater sociodemographic diversity in the images chosen for the site: “In terms of your photos, just make sure that there’s representation from across the board in terms of race, color, ability or disability, just to make it well-represented” (Participant PT).

Participants also identified information and resources that would make the website more relevant to TGD older adults. Participants were specifically looking for assistance finding affirming healthcare providers, learning about transitioning in later life, and becoming more informed about healthcare rights and policies, among other topics.

##### Design principles 1 and 2 (motivation and composition)

Participants also favored the inclusion of stories of TGD older adults with experience navigating the healthcare system as sources of advice and peer support in an uncertain sociopolitical climate:

I have a mentor now, he’s 65 and he’s teaching me how he has navigated his healthcare. Because…he’s 65, so he started hormones from the back alley. So, I’ve been listening to him to learn what it was like and how he addresses his healthcare providers now…that we seem to maybe be going, you know, a little more rigid (Participant TI).

##### Design principle 3 (interaction)

Recognizing risks to the TGD community posed by anti-transgender policies in the U.S., participants stressed the need for website security to protect TGD users, recommending the inclusion of a statement that user information would not be collected or shared with third parties.

In reviewing P2, Participant LH acknowledged technological barriers affecting some older adult users, and proposed the addition of a tutorial on the website:

I think having a how-to guide is probably really useful…Because, again, I’m just thinking about our experiences with a lot of our elder folks, and I use elder in a very loving way, but some of them are struggling just with navigating something like this.

#### Prototype 3

The third prototype (P3), another mid-fidelity model (Figure 2C), integrated participant feedback about the site’s layout and expanded on the site’s informational content. Informational content for P3 was composed by the PI (L. C.) from available evidence and was reviewed by three area experts in medicine, policy, patient advocacy, and transgender health. P3 included three information-based webpages with 77 frequently asked questions and one page dedicated to health-related stories of TGD adults in video, audio, and text formats. Design features, such as accordion dropdown menus, were added to organize information with simplicity and clarity according to participant preferences. Representative imagery, a site navigation guide, and a statement about data security were added.

### Testing phase

P3 was designed as a 10-page functional website suitable for testing by TGD older adults. Participant PT detailed the testing process:

The next step would be you want to pilot this within a small cohort of older adults who can then use [it] and see and then get their feedback…and then you’ll try to incorporate whatever feedback they have in terms of some of the loopholes or challenges that they might have experienced while using the site. After that, you are ready to launch it.

Sixteen participants who had never seen earlier prototypes of the website reviewed P3 during the testing phase. During the interview period, all 10 site pages were visited at least twice, and each participant viewed and assessed 5–6 pages on average (range: 2–9 pages). The most frequently visited pages contained links to resources (14 visits), health information and frequently asked questions (13 visits), and links to care provider directories (13 visits). The pages with the fewest visits were auxiliary pages with information about the site (2 visits) and site navigation (3 visits). Through the think aloud protocol, participants contributed over 200 affirmations of site usability and acceptability and just as many unique action items to enhance the site.

Three prototyping phase participants and 14 testing phase participants completed the SUPR-Q. Eight participants (47%) accessed the prototype on a mobile phone and 7 (41%) accessed it on a computer. Due to difficulties with connectivity and screen settings, two participants (12%) viewed P3 on their mobile device via the interviewer’s shared screen. Of the participants aged 50 and older, 70% accessed the prototype on a mobile phone. Usability and acceptability as measured in the SUPR-Q were rated favorably overall. The mean score for prototyping phase participants (*n* = 3) was 4.96 out of 5 total points; the mean score for testing phase participants (*n* = 14) was 4.52; and the mean score across both phases (*n* = 17) was 4.60. The credibility factor scored highest and appearance scored lowest (see Table 3). Figure 3 provides a graphical representation of testing phase SUPR-Q scores by factor in a joint display with associated qualitative feedback.

**Table 3:** Mean SUPR-Q scores and sub-scores by study phase.

Score	Prototyping (*n* = 3)	Testing (*n* = 14)	Both phases (*n* = 17)
Mean score	4.96	4.52	4.60
Usability sub-score	5.00	4.50	4.59
Credibility sub-score	4.83	4.61	4.65
Appearance sub-score	5.00	4.46	4.56
Loyalty sub-score	5.00	4.50	4.59

**Figure 3:**
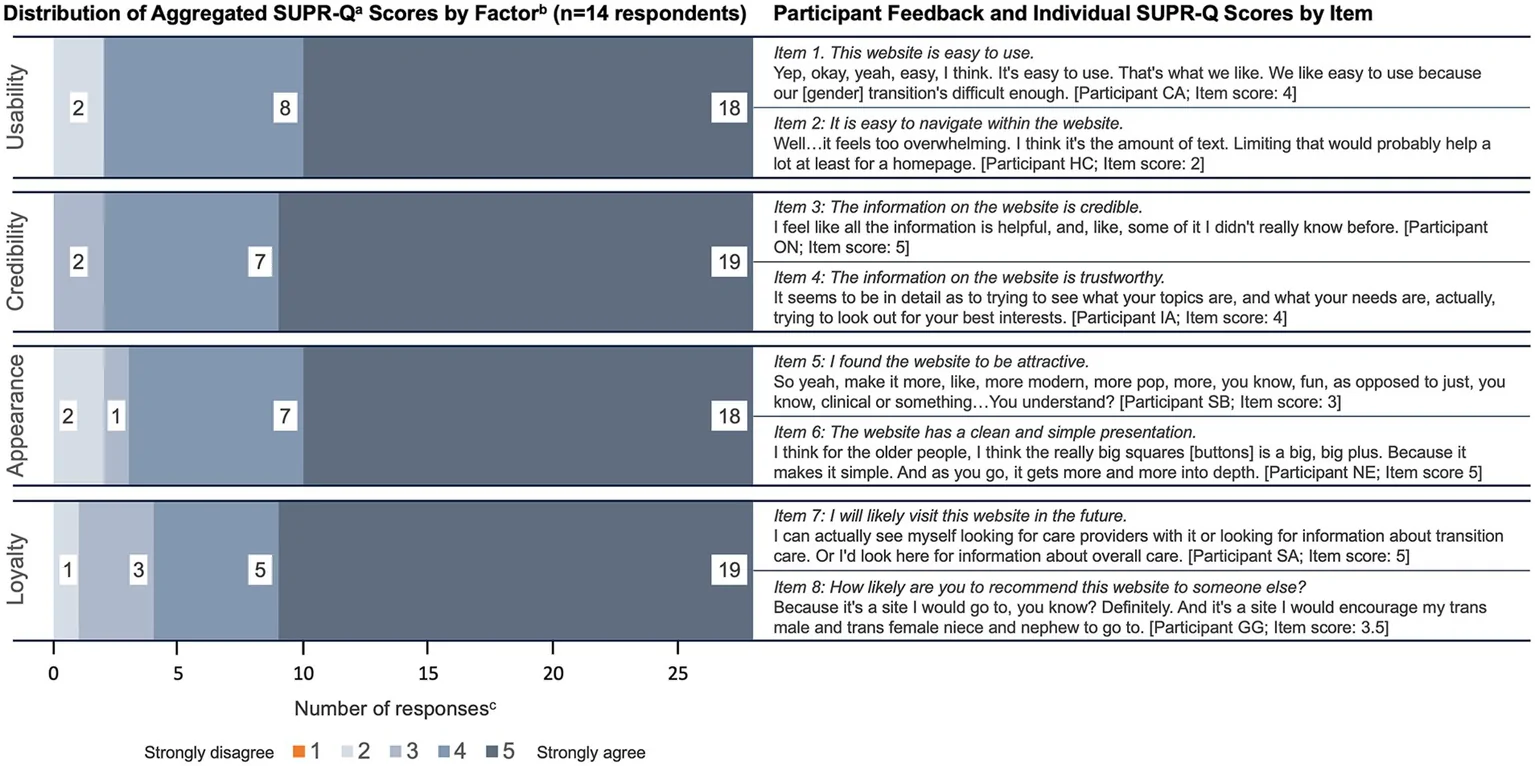
Joint display of testing phase survey results with qualitative participant feedback. ^a^Standardized User Experience Percentile Rank Questionnaire. ^b^Usability (Items 1 and 2): Ease of use and navigation; Credibility (Items 3 and 4): Perceived trustworthiness and safety; Appearance (Items 5 and 6): Attractiveness and simplicity of design; Loyalty (Items 7 and 8): Likelihood of returning to the website and recommending it to others. ^c^Participants each responded to two items per factor, so the total number of responses for each factor is 28.

#### Design principles 1 and 2 (motivation and composition)

Testing phase participants confirmed the suitability of the website for an audience of TGD older adults. Participant CA reiterated preferences for clarity and simplicity expressed by participants in the prototyping phase: “My age group likes simplicity. We don’t like to play hunt and seek.” Most participants acknowledged the potential for resources and stories linked on the site to help them as older TGD people. Participant EI underscored the importance of stories that represent healthcare experiences of TGD older adults: “That [story] would be cool, I’m gonna click on that just because when they talk about transgender care all the time on, even on progressive media…they never talk about transgender care for elders. It’s so hard to find information for elders.”

#### Design principle 2 (composition)

Participants recommended the addition of informational and visual content to promote site relevance and acceptability. Satisfaction with the representativeness of images on the site was mixed, with six participants overtly recommending greater racial and ethnic diversity. Participant GG called for greater representation of diverse racial and ethnic experiences while validating the goals of the website to promote inclusivity:

It’s important to have some informed knowledge of that sociopolitical and cultural nuance when you are not Black and trying to address issues that affect them…Because when I look at a website, I realize that you cannot possibly have all the answers for everybody, all at the same time, but the fact that you are even making an attempt, being transparent about it, being open about it, gives people hope.” [Participant GG].

Participants proposed 16 new subject areas to include on the site, such as social health, sexual health, immune health, and nutrition for TGD older adults, and expanded on existing topics like advance care planning. Speaking about social health, participant HC recommended including information to support people experiencing loneliness in later life, observing, “A lot of trans people are lonely, especially when they’re older, but even now there’s a loneliness…There’s a lot of lonely folks.” Participant TB drew attention to the need for nutrition information and resources as a form of illness prevention for older adults in later life, suggesting, “How about, some type of culinary [resource]? Because as we age, even as we get older, we need to start eating right and doing the right thing.” Participant JN voiced support for content about advance care planning “because everybody is aging, and as much as you want for people to be well their whole life and never get sick, or never have any challenges, we don’t get to control that.” He continued, “And you want them to have autonomy in their body for as long as possible”.

#### Prototype 4

From participant feedback in the testing phase, a high-fidelity prototype (P4) was developed for dissemination to the community (Figure 2D). Modifications to P3 were made based on participant feedback and usability survey scores aggregated by SUPR-Q factor (*usability, credibility, appearance*, and *loyalty*):

- To enhance *usability* and navigability, text density on the homepage was reduced, and dropdown menus were used throughout the site to limit the amount of text visible at any given time.
- *Credibility* was strengthened by listing essential transgender health resources that had been missing in earlier prototypes and by removing resources that participants deemed problematic. Images from a professional photographer were included to represent TGD older adults from diverse sociodemographic backgrounds. An additional 60 frequently asked questions and responses were incorporated.
- Changes to site imagery, interactivity, and icons were made to improve the *appearance* and appeal of the website.
- P4 maintained elements of earlier prototypes that were designed to promote *loyalty* such as offering relevant and up-to-date information about healthcare for TGD older adults in a format that is easy to share with peers and support persons. Notices about forthcoming content and features were added to encourage repeat site visits.

Supplementary material S3 displays a selection of participant contributions categorized by design principles and themes with examples of how each contribution was integrated into the site design. Figure 4 highlights selected participant contributions to the design of one page of P4 paired with the compositional features of the prototype that correspond to each contribution.

**Figure 4:**
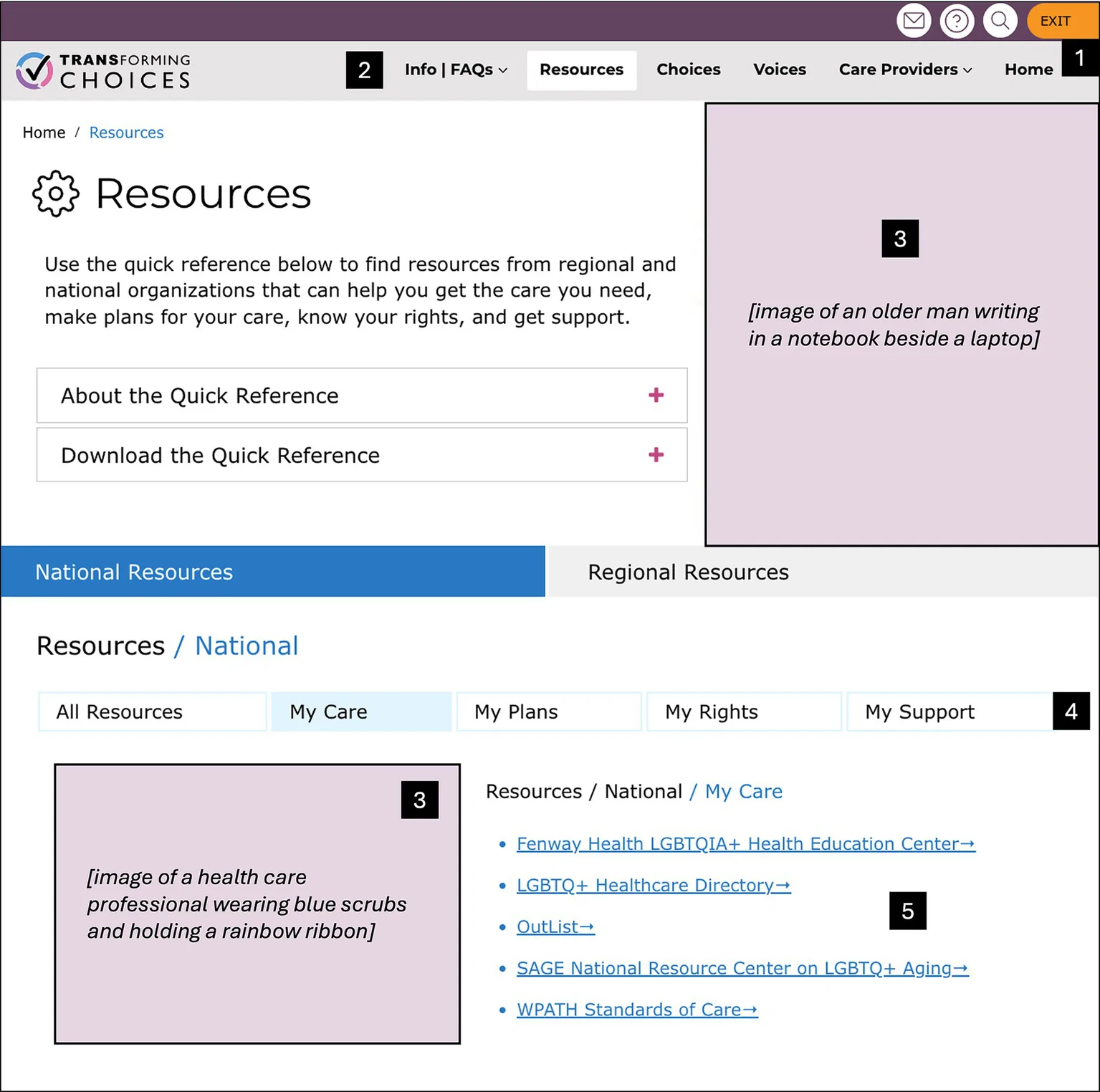
Joint display of a web-based health resource prototype and associated participant design suggestions. 1. EXIT BUTTON. The Exit button gives the user the option to leave the site quickly to protect their privacy: “I love that there’s an exit button for the site. That’s not something I was thinking about, but it’s definitely useful.” (Participant MW). 2. NAVIGATION MENU. The order of pages listed in the navigation menu follows an intuitive sequence and prioritizes topics with most relevance to users: “I think Information still should be number one because that will give you an insight. And then Resources then Choices.” (Participant CA). 3. SITE IMAGES. The site includes affirming images representative of TGD older adults and healthcare professionals from diverse backgrounds: “If you have trans femme, trans masculine people, and African American and European, Indigenous, and Hispanic…if you could see yourself in the [site] that would be helpful.” (Participant IA). 4. RESOURCES: FORMAT. The quick reference organizes resources by title and by category to aid users in searching the list: “Up top, you might want to say that some of these resources are healthcare-specific, and some are healthcare-supportive, and some are legal.” (Participant CZ). 5. RESOURCES: CONTENT. Resources on the site were verified for inclusivity, trustworthiness, and relevance to TGD older adults: “I had to teach my provider how to care for trans [people]…I’m her only trans patient. And Deutsch and WPATH were the best ones that I found.” (Participant JM).

### Rollout

Following a rapid review process, P4 was disseminated as a fully functional website to a selection of individuals and organizations with an interest in healthcare for TGD older adults. Dissemination was carried out in phases to avoid premature expansion without proper infrastructure and to reduce the risk of exposure to anti-transgender responses. Dissemination of this resource followed a plan for ongoing outreach in line with the larger vision participants articulated for expanding it. From the list of ideas related to resource expansion and sustainability, the research team developed a progress plan that was reviewed and refined by participants. This progress plan summarizes action steps to disseminate, expand, and sustain the resource over time. For example, the progress plan covers options to promote sustainability through grants, partnerships, a community advisory board, and intervention development or nonprofit formation. An excerpt from the progress plan can be found in Supplementary material S4.

To view the web-based resource developed in this study, visit Transforming Choices [https://www.transforming-choices.org].

## Discussion

In this multimethod study, participants and researchers used a human-centered design approach to co-design a web-based health resource for TGD older adults. From thematic analysis of data from semi-structured interviews, the research team developed four resource prototypes of increasing fidelity guided by five design principles and themes: (1) Motivation: Facilitating healthcare decisions through information and resources; (2) Composition: Organizing complex content with clarity and inclusivity; (3) Dissemination: Phasing rollout to communities and organizations; (4) Interaction: Maintaining security, accessibility, and navigability; and (5) Expansion: Planning for leadership, stewardship, and partnership. The research team prompted design recommendations from participants during their interviews using a think aloud approach. Prototypes were developed by the research team in consultation with community advisors from a descriptive analysis of participant recommendations and responses to a website usability survey.

Participants in the prototyping phase and earlier phases of the SAFEage study emphasized the need for responsive website design to make the site accessible to TGD older adults using any device (i.e., computers, tablets, and mobile phones). Seven of 10 older adult participants accessed the third prototype using a smartphone, exemplifying the rise in mobile device use among older adults (30). Testing phase participants also reported a high level of comfort with using websites, which suggests a higher degree of digital literacy (the ability to effectively use digital technologies) in this group of TGD older adults than may be expected in the general population (31). Given the objectives of the final two phases of the SAFEage study to prototype and test a web-based resource, it is likely that participants with higher interest and skill with technology self-selected into the study. Nevertheless, participants were mindful of their peers with limited digital literacy and sensory disabilities, advocating for a simple, user-friendly design for the resource with user tutorials and intuitive navigation. The accessibility of future iterations of the website for TGD older adults may be enhanced by integrating participant recommendations and current evidence for accessible web design features, including user support, controls for color contrast, optimization for screen readers, and other features (32).

In the testing phase, SUPR-Q scores for the third prototype differed by factor. The site’s appearance was rated the lowest of the four factors (4.46 out of 5) among testing phase participants. Based on qualitative feedback from participants, appearance received a lower rating likely due to the use of imagery and a color palette that participants deemed too “clinical” and not sufficiently engaging. Consequently, in the design of the fourth prototype, the research team included more LGBTQIA+ oriented imagery and color choices and added interactive features on the homepage and on several subpages. Of note, the highest SUPR-Q factor score among testing phase participants was for credibility (4.61 out of 5), which may reflect participants’ perception of the resource’s relevance to their own experience and their satisfaction with the usefulness of sources and resources listed on the site. The research team strengthened site credibility in the fourth prototype by adding citations of current evidence, expanding the range of content areas, and incorporating expert reviews from clinicians, TGD older adults, and others with relevant knowledge and lived experience.

Representation of diverse gender identities and races and ethnicities within the TGD community was essential to the integrity of the SAFEage study. Representation of diverse gender identities in the study is consistent with the distribution of gender identities in the U.S. TGD population, albeit with a larger percentage of participants who identify as gender diverse (3). By race and ethnicity, the participant group had a lower proportion of White participants than estimates of White TGD adults in the U.S.; concomitantly, the proportion of people of color represented in the participant group was higher than national estimates (3). High engagement from participants of color led to improvements in each resource prototype that increased the usability and relevance of the resource for racially and ethnically diverse users. For example, based on participant suggestions, links to services and organizations serving Black, Asian, and Hispanic trans people were added to the site’s resources page, and information about gender-affirming care for trans people of color was expanded. Notably, recommendations to promote equitable representation of people of color in the resource came from both White and BIPOC (Black, Indigenous, People of Color) participants.

A growing number of studies have used co-design and HCD approaches to engage TGD youth and younger adults in health research. For instance, researchers working with TGD participants have designed tools to aid TGD youth and young adults in shared decision-making about gender-affirming medical care through iterative co-creation workshops (33, 34). Researchers have also used a web-based participatory design process to engage TGD young people in designing health information resources (35, 36). Findings from these studies were based on data from predominantly younger (under 50 years old), White participants. The SAFEage study expands on existing research by centering engagement with older adults and people of color, who have been historically underrepresented in co-design research (20, 37).

The body of co-design research on digital health for LGBTQIA+ adults is limited and has not focused specifically on TGD older adults in the U.S. (36, 38, 39) By reporting on the prototyping and testing phases, this study helps fill a gap in the literature on co-design for older adults (40) while forming a design framework to facilitate future health research that engages the TGD community. Further research is warranted to test the effectiveness of the health resource developed in this study, measuring outcomes such as health knowledge and self-efficacy, ACP engagement, and social connection among TGD older adults.

Study findings have clear implications for public health research, practice, and policy. Merging topics in technology, aging, and health, this study lays the groundwork for future community-based, culturally inclusive digital health interventions to facilitate informed healthcare decision-making among TGD older adults. Participant suggestions for methods to promote resource sustainability and scalability are covered in the progress plan co-written by study contributors as a guide for resource expansion. Expansion of this study will center on community integration through reinforcing ownership of the resource by community members; increasing acceptability of the resource among TGD older adults from culturally diverse backgrounds; and forming a community advisory board to drive intervention development. Aligning growth with existing initiatives and policies will be essential (41). As an evidence-based digital resource, the website generated during the study may be used as an accessible tool for health education for TGD older adults across the U.S. and as a way to disseminate credible public health messaging that is relevant to the TGD community. Moreover, the website includes information and resources for TGD older adults, clinicians, and caregivers to learn about national policies affecting healthcare for TGD older adults and to advocate for equitable care for TGD older adults at the institutional and governmental levels.

### Limitations

The SAFEage study has methodological and practical limitations. The study employed a descriptive approach to both qualitative and quantitative analyses; thus, no theoretical interpretations or statistical inferences were made from the data. Although this approach limits generalizability, the range of perspectives in the data support transferability of the findings to related contexts. While representativeness of the participant group strengthens transferability of the findings, the study is limited by the absence of Latino/a/e participants from the prototyping and testing phases. Future outreach would require further rapport-building and community engagement that is specifically tailored to Latino/a/e adults. Moreover, while the quantity and depth of data in this study were strong, data originated from a small to moderate size participant group, so additional perspectives relevant to the study purpose may have been missed.

Because SUPR-Q responses were primarily elicited in real time during participant interviews, social desirability response bias is a possible limitation, despite prompting by the interviewer to encourage honest responses. Consequently, it is possible that the mean SUPR-Q score overestimates participant satisfaction with prototype usability. Also, the fact that prototyping phase respondents were co-creators of the resource may have influenced the high mean score observed in this phase, which in turn inflated the mean score across both phases. Thus, the mean score for both phases should be interpreted with caution. Furthermore, due to concerns about participant burden and low response rate, the SUPR-Q was not readministered to obtain scores for the fourth prototype, which limits assessment of its improvement over the third prototype. However, qualitative responses from a subset of participants and collaborators after the preliminary release of the fourth prototype were favorable and no substantial changes were recommended at that time, suggesting high satisfaction with the resource.

## Conclusion

As one of few studies to use a human-centered design approach to develop a health resource with TGD older adults, this study demonstrates innovative methods for addressing inequities affecting this population. Through an iterative, participatory process, TGD adults and older adults of diverse gender identities and from diverse racial and ethnic backgrounds participated alongside researchers and multidisciplinary professionals in designing a web-based health resource to benefit their communities. As part of this process, a set of five design themes was generated to guide resource development. The resultant website addresses gaps in access to information and resources for TGD older adults to support informed healthcare decision-making and serves as a tool for use in future community-based intervention research.

## Data Availability

The datasets presented in this article are not readily available because Data have not been approved for sharing beyond the original study. Requests to access the datasets should be directed to Lauren Catlett, lec2c@virginia.edu.
