# Supplementary material for: Co-designing a digital health resource for transgender and gender diverse older adults
Source: Front Public Health. 2026 Oct 28;14:1919463. doi: 10.3389/fpubh.2026.1919463 (PMC13751331; doi:10.3389/fpubh.2026.1919463)
Supplement: Supplementary file 1 [file Data_sheet_1.pdf]

Prototyping Phase, Initial Interview Guide

[Introduction]

Opening Questions
- To start, please tell me a little about yourself. What is important for me know about you?
- *[For returning participants]* To start, please share anything that has come up for you related to your health or your healthcare since we talked last. What is important for me to know?

Web-based Prototype 1 (Low-fidelity) *Generative Techniques* (Melles et al., 2021)
[Show slideshow of images and ideas generated from earlier phases of the study]

Content *[Review features, content areas, topics; Ask for questions]*
- Looking at this list, what features or topics would you add? Which would you take away?

Layout *[Review features and specifics; Ask for questions]*
- What else would be important for the layout?

Accessibility *[Review accessibility topics; Ask for questions]*
- How else could we make the resource accessible to trans and gender diverse (older) adults?

Sustainability *[Review sustainability topics; Ask for questions]*
- What would be important for maintaining and expanding on the resource over time?

Homepage Prototype *[Show a mock-up of a website homepage based on the features discussed earlier]*
- What are your thoughts about this mock-up? What would you keep? What would you take away?

Next Steps
- Based on your experience, what would be important to include in this model to make the resource useful for trans and gender diverse (older) adults? What would make it accessible?

- What else would you like me to know before we finish up? Is there anything you'd like to add that I didn't ask about?

[Conclusion]

References
Melles, M., Albayrak, A., & Goossens, R. (2021). Innovating health care: Key characteristics of human-centered design. *International Journal for Quality in Health Care*, *33*(Supplement_1), 37–44. https://doi.org/10.1093/intqhc/mzaa127

Prototyping Phase, Follow-up Interview Guide

[Introduction]

Web-based Prototype 2 (Mid-fidelity)
[Show prototype homepage] Take a moment to look it over and as you do, let me know what you're thinking. Also let me know when to scroll down.

As a person just coming onto this site:

- What is your first impression?
- What message(s) are you getting?
- Can you tell what this website is for? How?
- How easy would it be to navigate to another page?
- Would you stay on this site to learn more?
- What works well here?
- What needs improvement?

Where should we go next from the homepage? What would you like to see?
[Show page of participant's choosing and invite discussion]

[Show 1-2 more webpages and invite discussion]

After seeing this model, what would make it more appealing?

Before we talk about future plans for the website, what else would you like to share about the model we just looked at?

Web-based Prototype Future Plans

- Having seen this model for the website, what do you see as our next step toward developing the model into a resource to share with the community in the short term?

- What would be your vision for this resource in the long term?

- How could this resource be expanded holistically to address needs and challenges facing transgender and gender diverse adults at all levels (individual, community, and society)?

- How can we get the resource to people who need it?

- What challenges do you foresee and how could they be addressed?

What else would you like to share? Is there anything we didn't cover?

[Conclusion]
