# Supplementary material for: Co-designing a digital health resource for transgender and gender diverse older adults
Source: Front Public Health. 2026 Oct 28;14:1919463. doi: 10.3389/fpubh.2026.1919463 (PMC13751331; doi:10.3389/fpubh.2026.1919463)
Supplement: Supplementary file 2 [file Data_sheet_2.pdf]

Testing Phase Interview Guide

[Introduction]

Web-based Prototype 3 (Mid-fidelity)

<u>Orientation</u>
[Provide background information about the prototype (name, web address, how it came about, etc.)]

<u>Prelude</u>
The goal of having you use this website is to see how well, or poorly, it works. Please give your honest feedback about the website. The more open you are with feedback, the more improvements we can make.

[Ask] **What device will you be using to view the website?**

[Send prototype link and password (if applicable) to participant]

<u>Homepage</u>
Take some time to look at what's on the homepage. As you do that, please say what you're thinking, what you're looking at, and what you're trying to do ***out loud***. This will help me understand the site from your perspective. *[Invite participant to share their screen, if applicable]*

[Participant navigates the home page and engages in think aloud process] (LUMA Institute, 2019)

[Ask] **Does the homepage make you want to see what else is on the site? Why or why not?**

<u>Scenarios</u>
Choosing from the options in the menu bar at the top of the page, where would you like to go next? Please tell me aloud how you'd get there. *[Participant chooses a webpage prototype to visit next]*

<u>Scenario 1: Example Subpage 1</u>

1. What kinds of topics do you think you'd find under the headings [X, Y, or Z]?

2. If you're looking for more information about each of these headings, how could you find out without leaving this page?

3. Choose one of the headings and navigate to the corresponding page. Tell me how you got to the next page and what you see when you're there.

4. How would you find information about [X]?

5. How useful would this page be for you?

Scenario 2: Example Subpage 2

1. What is the purpose of this page? How do you know what it's for?

2. How would you download content from this page?

3. What do you wish to see on this page that you don't see here?

4. How useful would this page be for you?

Scenarios 3-4: *[Participants choose subpage to view]*

Scenario 5: Let's say you wanted to reach out to the organizers with a question or comment. How would you do that? Please share the steps you take out loud.

Scenario 6: Let's say you wanted to know what the site icons mean or how to make the text size bigger on the site. Where on the site could you find help for that?

[Ask] **How would you get back to the homepage where we started?**

General Feedback
What were your overall impressions of the website?

How easy or difficult was it to use the website?
What did you like about it?
What would you change about it? What could make it better?
What is missing? What would make this website more useful?

How helpful would this website be in making you better informed to make decisions about your healthcare?

Could you see yourself using this resource in the future? Why or why not?

What else would you like to share about the website or anything we talked about today?

[Conclusion]

References
LUMA Institute. *Innovating for People: Handbook of Human-Centered Design Methods*. Pittsburgh, Pennsylvania: LUMA Institute; 2019.
