# Supplementary material for: Co-designing a digital health resource for transgender and gender diverse older adults
Source: Front Public Health. 2026 Oct 28;14:1919463. doi: 10.3389/fpubh.2026.1919463 (PMC13751331; doi:10.3389/fpubh.2026.1919463)
Supplement: Supplementary file 3 [file Data_sheet_3.pdf]

PROGRESS PLAN //

TRANSFORMING CHOICES 2025 and beyond

This plan outlines goals and action steps for developing **TRANS**FORMING CHOICES, a web-based healthcare decisions resource for transgender and gender-expansive adults and older adults. The plan covers the vision and purpose of the resource as well as strategies for outreach, sustainability, and growth over time.

Ideas for this plan were contributed by over thirty collaborators involved in the research project *Shaping Affirming Futures with Elders of all gender experiences* (SAFEage). Collaborators included trans community members, healthcare professionals, designers, aging services providers, and others of diverse gender identities and racial and ethnic backgrounds.

REVIEW COPY

Prepared in 2026 by Lauren Catlett, PhD, RN; Ames Simmons; April D.J. Snow; B.C.; C.C.; Charley Burton; Crystal D. Chu, RN, PhD; G.M.B.; Jay L. Austin; Jen Turpen, DO; Kylie Madhav; Noelle Marie Javier, MD; Vann Millhouse, B.S., M.A.

With support from the American Psychological Foundation and the University of Virginia Compassionate Care Research Pilot Fund Award

Copyright 2026 © Lauren Catlett

TABLE of CONTENTS //

Core Concern-- 3

Vision-- 4

Purpose-- 5

Site Features--- 6

Future Features-- 7

Outreach Framework--- 8

Maintenance Framework-- 13

Protections--- 19

Support-- 21

Timeline--- 25

REVIEW COPY

CORE CONCERN //

Transgender and gender-expansive adults and older adults need accessible, gender-inclusive resources to help them mitigate current health challenges and prepare for future care because many:

- Face injustices on the basis of age, race, ability, and/or gender identity, contributing to poor health outcomes.
- Have limited access to affirming healthcare services due to anti-transgender policies and ineffective health systems.
- Lack adequate social and medical resources and support for their care in later life or during a serious illness.

In addition, many trans and gender-expansive adults lack reliable information to make choices within the healthcare and insurance industries, and they often distrust (or need an alternative to) their current providers due to anti-trans political shifts.

"I think especially now, it's going to become, I mean, truly, a life-saving thing, right? As you know, as the world continues to just fall apart with respect to trans rights, especially in this country, I think this is going to be something that a lot of folks get a lot of benefit from."

REVIEW COPY

VISION //

We envision a future for healthcare in which transgender and gender-expansive adults and older adults:

1. Feel safe in healthcare spaces;
2. Are treated with respect by healthcare professionals and staff;
3. Advocate for themselves and exercise agency in their healthcare decisions;
4. Find people to support them and advocate for them in healthcare settings if they are unable;
5. Receive respectful and inclusive care from trained healthcare providers;
6. Access gender-inclusive health services and gender-affirming treatments with fewer obstacles;
7. Find resources for healthcare irrespective of age, stage of health, or gender expression;
8. Feel confident that they can find answers to general health- and transition-related questions.

"Based on what I've heard from patients, is that…recognizing which places would be inclusive and safe, is really important. And so, I don't know what kind of information you could link to that, but I know that that's really important when they're considering where they're going to go for their care and what access to care they have."

REVIEW COPY

PURPOSE //

The purpose of **TRANS**FORMING CHOICES is to help transgender and gender-expansive adults and older adults:

1. Make informed healthcare decisions now and in later life;
2. Access healthcare information, resources, and support.
3. Communicate their health care wishes and decisions to people in their support networks and healthcare professionals they trust.

REVIEW COPY

"I think this is the sort of thing that needs to be out there right now. I mean, particularly right now, when you think about all that's gonna be happening, chaos-wise, over the next four years, probably to have something that's just an objective, informational resource that cuts through all the noise and says, 'Here's where you can get help. Here's where you can find others who've been through this.'"

SITE FEATURES //

TRANSFORMING CHOICES provides information, resources, and support in the form of a website transforming-choices.org. Key features of the site include:

CONTENT

- Answers to frequently asked questions about healthcare, healthcare decision-making, and healthcare rights
- **TRANS**FORMING CHOICES Healthcare Decisions Starter Guide
- Links to stories and testimonials
- Resources on advance care planning, healthcare, long-term care, health policy, and more
- Information and resources for care providers
- Guidance for using the site and associated resources
- Contact form and email to submit questions and feedback

DESIGN

- User-friendliness for adults of all ages
- Images representing people from diverse backgrounds
- Accessible design for people with specialized viewing needs
- English and Spanish translations of site content
- Site security and regular maintenance
- Responsive design for use on PCs and mobile devices
- Optimized for discovery by search engines

"I think this idea of the first thing that you see can't be too complicated, because you don't want to overwhelm people immediately. They're coming in with no idea of what to expect. But at the same time, you want to have enough information that they know what to do from there."

REVIEW COPY

FUTURE FEATURES //

Depending on available support and resources, the following features may be added to the site in the future:

CONTENT

- Additional downloads and infographics
- Interactive tools to facilitate healthcare decision-making
- Searchable directory of national and regional resources
- Forum for trans community members and healthcare professionals
- Group and individual virtual meetings with care navigators
- Phone number to reach care navigators and to get site help
- Social media pages to connect with the community
- Education and certification for healthcare providers
- Internal healthcare provider directory

DESIGN

- Integration of AI features for user assistance
- Development of a TRANSFORMING CHOICES app
- User accounts to save and store user-generated content
- French and Mandarin translations of site content

"I only think sometimes it's nice, I don't know if this is possible, if it makes sense, if people can create their own kind of profile without fear of it being out there, stolen or something. But, and then, like, 'Here's the information, like, things that I looked at and I want to save those and come back to them.' So, perhaps when you come back to the website, you don't have to do everything again."

REVIEW COPY

References

Acquaviva, K. (2023). The Handbook of LGBTQIA-Inclusive Hospice and Palliative Care. Columbia University Press.

Catlett L, Simmons A, LeBaron V, et al. Co-designing an advance care planning resource with transgender and gender diverse adults. *Design for Health.* 2025;0(0):1-20. doi:10.1080/24735132.2025.2561340

Catlett L, Simmons A, Williams IC, et al. Multilevel Factors Affecting Health Equity for Transgender and Gender Diverse Older Adults. *Qual Health Res.* Published online June 11, 2025. doi:10.1177/10497323251342162

Simmons A. Advance Directives to Manage Fears and Anxieties of Transgender People via Dementia Planning. *J Law Med Ethics*. 2024;52(3):704-716. doi:10.1017/jme.2024.136

REVIEW COPY

Please contact Lauren Catlett (LEC2C@virginia.edu) to inquire about the full document
