# Supplementary material for: Co-designing a digital health resource for transgender and gender diverse older adults
Source: Front Public Health. 2026 Oct 28;14:1919463. doi: 10.3389/fpubh.2026.1919463 (PMC13751331; doi:10.3389/fpubh.2026.1919463)
Supplement: Supplementary file 4 [file Data_sheet_4.docx]

**Supplement 4.** Participant contributions to resource development by design theme with examples of website integration

| **Design Principle: Theme** | **Participant Contribution** | **Integration into Site Design** |
| --- | --- | --- |
| Motivation: Facilitating healthcare decisions through information and resources | I kind of have a thing for trying to assemble, like, hubs of information. There’s a lot of information that already exists. And there's no sense in doing more work than you have to if everybody else has already made the resource, right? (Participant MW) | The following subtitle was added to the website homepage: “An information and resources hub to support empowered healthcare choices now and in later life for transgender and gender-expansive elders, younger adults, and care providers.” |
|  | It makes it feel like you have some agency in this. I am empowering myself because this is the information I was seeking. (Participant LH) |  |
| Composition: Organizing complex content with clarity and inclusivity | I like the layout. It's soothing. It's not so in depth that you're getting inundated with any one thing. I think you can figure out where you want to look at next without having information overload. (Participant CB) |  |
|  | I also liked how every page has a picture of a person on the top right, and I think they were diverse. And, well, I don't know if every page has this, but they represented the trans community. (Participant SA) |  |
| Dissemination: Phasing rollout to communities and organizations | You may have to start somewhat backwards, you know, like, are there care providers that would be able to tell people, if somebody is their patient, and say, ‘Well, you're reaching this age and you need to make some decisions. And here's some tools that can help you think about how you want to make those  decisions.’ (Participant AK) | In the year following release of the website, outreach plans included five presentations to healthcare providers and aging services providers at professional conferences and meetings across the United States. |
| Interaction: Maintaining security, accessibility, and navigability | If the site is collecting anything about people, maybe you have some disclaimer that just reminds people that, you know, I don't know, but I just know that there's a level of concern that people have been expressing about just exposing themselves in any kind of form. (Participant LH) | The following disclaimer was added to the Contact page of the website: “We do not collect or share personal identifying information.” |
|  | Make [site pages] readable. Make them, you know, for folks like me, who now use readers, you know. Make them so that those of us who have a little bit of limited visibility or vision can navigate through without having to read mouse-print down at the bottom. (Participant CD) | The font size selected is larger than average:   |
|  | I would need an explanation or directions, actually, because I wouldn't have found it on my own if you hadn't told me. (Participant IA) |  |
| Expansion: Planning for leadership, stewardship, and partnership | Because this is going to be something, this is going, this is literally a living document. It's going to continue to change and look different all the time as we evolve and change. (Participant DK) |  |
